# A Case of Chronic Urithritis Treated by Emmett’s Button-hole Operation

**Published:** 1886-07

**Authors:** Virgil O. Hardon

**Affiliations:** Atlanta, Ga.


### Clinic Reports.

### A CASE OF CHRONIC URETHRITIS TREATED BY EMMET'S BUTTON-HOLE OPERATION.

By VIRGIL O. HARDON, M. D., Atlanta, Ga.

E. J., white, widow, aged 61, was married at 13 and has borne 19 children. All her labors were normal, as far as she knows, and her health has always been good until twelve years ago. She then began to suffer from frequent desire for micturition, and the act was always accompanied by burning pain. These symptoms gradually increased in severity until at the present time she is obliged to urinate at intervals of from fifteen to thirty minutes throughout the day and night. The passage of urine produces an intense pain in the urethra, especially at the meatus, radiating upward into the abdomen and downward into the thighs. This pain persists for some time after micturition, so that she is hardly ever free from it. In other respects her health is good, but her naturally robust constitution is breaking down under the constant pain and annoyance to which she is subjected. She is entirely unfitted for social or domestic duties, and nearly her whole time and attention are given to keeping her bladder empty.

Examination shows the meatus contracted so as to scarcely admit a No. 6 sound, and surrounded by cicatricial tissue, forming bands by which it is much distorted. Extreme tenderness exists along the urethra and in the neck of the bladder. The passage of a sound gives exquisite pain. The urethro-vaginal septum is of abnormal thickness and density. Otherwise the pelvic organs are found to be normal.

The urine, of which about an ounce is passed at a time, is straw-colored and slightly turbid. Upon standing there is formed a deposit of about one-fourth its bulk. Specific gravity, 1028. Chemical and microscopical examination shows it to be free from albumen, sugar, pus and mucus. The deposit is made up of amorphous urates.

The patient has been treated by internal medication by competent practitioners, but without receiving any apparent benefit.

January 23, 1886, with the assistance of Drs. Bizzell and Wile, she was etherized and Emmet's button-hole operation was performed. An incision was made through the urethro-vaginal septum, commencing a quarter of an inch behind the meatus and extending to a quarter of an inch from the neck of the bladder. Through this opening the cut edge of the urethral mucous membrane was drawn and stitched on all sides to the cut edge of the vaginal mucous membrane with carbolized silk sutures. Thus no surface was left uncovered to heal by granulation. The urethral mucous membrane was found to be so intensely congested as to present a deep purple color and the capillary oozing of blood from it was very free. The parts were smeared with vaseline, and the patient was afterwards instructed to make the same application before each micturition. The wound healed satisfactorily and the sutures were removed on the eighth day, leaving a permanent urethro-vaginal fistula.

In the twenty-four hours following the operation, the patient urinated five times with only slight pain. After the second day she was entirely free from pain and has continued so ever since. She urinates sometimes twice, usually only once, and occasionally not at all during the night, and from four to six times during the day. She frequently holds her urine for six hours without any discomfort. The urine passes entirely through the artificial opening. The pain at the meatus and the tenderness along the urethra have ceased, and the congestion of the urethral mucous membrane is now very slight.

